# Associations between the standard deviation of apparent temperature and sudden cardiac arrest during the Taipei Marathon, 2013–2024

**DOI:** 10.3389/fphys.2026.1843910

**Published:** 2026-09-18

**Authors:** Yin-Hsien Lin, Chun Chen, Shang-Lin Hsieh, Pang-Yen Chen, Tsu-I Hsia, Chorng-Kuang How, Yen-Kuang Lin, Yu-Hui Chiu, Kuo-Song Chang, Ming-Kun Huang, Ding-Kuo Chien, Wen-Han Chang

**Affiliations:** 1 Department of Emergency Medicine, MacKay Memorial Hospital, Taipei, Taiwan; 2 Department of Artificial Intelligence and Medical Application, MacKay Junior College of Medicine, Nursing, and Management, Taipei, Taiwan; 3 Institute of Biomedical Engineering, National Tsing Hua University, Hsinchu, Taiwan; 4 School of Medicine, College of Medicine, MacKay Medical University, New Taipei City, Taiwan; 5 Graduate Institute of Life Sciences, National Defense Medical University, Taipei, Taiwan; 6 Department of Emergency Medicine, Taipei Veterans General Hospital, Taipei, Taiwan; 7 Department of Emergency Medicine, School of Medicine, National Yang Ming Chiao Tung University, Taipei, Taiwan; 8 Graduate Institute of Athletics and Coaching Science, National Taiwan Sport University, Taoyuan, Taiwan; 9 Department of Emergency, School of Medicine, College of Medicine, Taipei Medical University, Taipei, Taiwan; 10 Graduate Institute of Long-Term Care, MacKay Medical University, New Taipei City, Taiwan; 11 Department of Electronic Engineering, National Taipei University of Technology, Taipei, Taiwan

**Keywords:** apparent temperature, bystander-initiated CPR, emergency medical services, marathoner, mobile AED, sudden cardiac arrest, temperature variability

## Abstract

**Background:**

Climate change has led to a discernible increase in the intensity of extreme weather events, contributing to growing public health concerns regarding temperature-related mortality and morbidity. Recently, there has been an increase in the number of sudden cardiac arrests (SCAs) occurring during annual Taipei Marathon events compared with previous years. This study aimed to explore the correlation between weather conditions and SCA risk in runners.

**Methods:**

This retrospective observational study used data from the Taipei Marathon event held between 2013 and 2024 in Taiwan. We examined the relationship between the SCAs and weather conditions for each year using a Poisson regression model.

**Results:**

Among the 95,866 full- and 220,580 half-marathoners, all SCAs occurred in Taiwanese runners. There were three SCAs in male marathoners (median age, 50 years). In half-marathon runners, 10 SCAs occurred (median age, 30 years), including nine males and one female. Each 1 °C increase in the standard deviation of apparent temperature (AT) over the 7-day time window (lag 0–6) was associated with an SCA incidence rate that was 6.63 times as high on race day (IRR = 6.63, 95% profile likelihood CI 1.85–52.11; LRT p < 0.001). The incidence rate was significantly higher during half-marathons than during full marathons (4.53 versus 3.13 per 100,000 participants) and among men than among women (5.17 versus 1.18 per 100,000). The highest proportion of SCA (61.5%) occurred in the final quarter of the race. Bystander-administered cardiopulmonary resuscitation (CPR) was provided in all SCA events (100%). Automated external defibrillator shocks were delivered to 11 of 13 runners (84.6%). All 13 runners (100%) achieved return of spontaneous circulation in the field.

**Conclusion:**

Greater short-term AT variability was associated with a higher incidence of SCA during Taipei Marathon events, suggesting that temperature variability over several days preceding and including race day may be associated with SCA occurrence. Runners should remain highly vigilant and take proactive measures under cold/high-risk weather conditions. The on-site presence of trained first responders to initiate CPR is mandatory at these events. A well-planned model of in-event emergency medical services is of vital importance in scenarios such as marathon events.

## 1 Introduction

Large-scale mass-sporting events pose unique challenges for emergency health teams. Cardiac arrest in marathon runners is most associated with underlying cardiovascular disease, as the prolonged exertion and cumulative physiological stress become particularly pronounced during the later stages of the race (Day and Thompson, 2010, Kim et al., 2012). Marathon running requires prolonged high levels of aerobic metabolism (approximately 70–90% of VO_2_max), which places substantial demand on the cardiovascular system (Costill, 1972; Joyner, 2017). Furthermore, exposure to cold weather may act as a triggering factor by increasing cardiovascular strain during prolonged endurance exercise. No and Kwak demonstrated that submaximal exercise performed by soccer players in a cold environment (10 °C) elicited higher oxygen consumption, heart rate, and ventilation compared with exercise performed in a temperate environment (22 °C) (No and Kwak, 2016). A recent nationwide study involving approximately 4.53 million runners in Japan demonstrated that marathon races with reported sudden cardiac arrests (SCAs) occurred more frequently in winter and lower air temperatures, although a less pronounced increase in SCA risk was also observed at higher temperatures (>15 °C). Lower air temperatures at the start of a marathon were linked to an increased risk of SCA during races, with each 1 °C decrease associated with an approximately 5–6% increase in risk (Kato et al., 2026).

In addition to absolute air temperature, short-term temperature variability in the days preceding a marathon may also influence cardiovascular risk on race day. Although the mechanisms linking short-term temperature variability to exercise-related SCA have not been fully clarified, acute temperature fluctuations may affect cardiovascular physiology through changes in heart rate, blood pressure, blood coagulability, peripheral vascular tone, and autonomic cardiac regulation (Keatinge et al., 1984; Guo et al., 2016; Hanneman et al., 2026). Donaldson et al., although not specifically investigating athletic populations, reported a lagged association between cold exposure and ischemic heart disease mortality among individuals aged > 50 years in South East England, with the excess risk peaking approximately 3 days after exposure to temperatures from 0 to 15 °C (Donaldson and Keatinge, 1997). Similarly, Zhang et al. reported that greater short-term temperature variability (lag 0–7 days) was associated with an increased risk of all-cause mortality across China, the United Kingdom, and the United States, with stronger associations observed in populations living in warmer locations (Zhang et al., 2019). However, the association between short-term temperature variability and exercise-related SCA during mass-participation endurance events remains unclear.

The Taipei Marathon attracts approximately 30,000 participants each winter in a subtropical region, yet SCAs occur almost every year (Hsia et al., 2023). It comprises full- and half-marathon races. Between 2013 and 2024, SCA occurred in 13 Taiwanese runners among a total of 316,446 participants (4.11 cardiac arrests per 100,000 runners). Notably, the 2021 and 2023 Taipei Marathons recorded higher numbers of SCAs than the annual events from 2013 to 2020 (1.9 per 10,000 participants in 2021 and 1.1 per 10,000 participants in 2023) (Hsia et al., 2023). Although climate change has led to generally warmer winter conditions in Taiwan, considerable temperature variability and intermittent cold extremes remain common (Wang et al., 2010; IPCC, 2021). Given the timing of the event, participants may be exposed to substantial fluctuations in air temperature in the days preceding and during the race. These observations have raised concerns regarding the potential influence of weather conditions on cardiovascular events during endurance exercise. As apparent temperature (AT) integrates air temperature with additional environmental factors influencing thermal perception and physiological strain, it may provide a more physiologically relevant measure of environmental exposure during exercise than air temperature alone. In this study, we assembled a database spanning 2013–2024 to examine whether the standard deviation (SD) of AT on the days preceding each Taipei Marathon event was associated with the incidence of reported SCAs on the corresponding race day, and to describe the emergency medical services (EMS) provided to affected participants.

## 2 Materials and methods

### 2.1 Sources

The Taipei Marathon event takes place annually in December in Taiwan (25°02’15″ N latitude, 121°33’45″ E longitude). The full marathon route has remained consistent over the years: it begins at Taipei City Hall, proceeds along municipal roads, passes through the inner and outer streets along the Keelung River, and then returns to the municipal roads before finishing at the square in front of the city hall (the finish line was at Taipei Athletic Stadium in 2013 and 2014) (**Figure 1**). The half-marathon course shared approximately 70–80% of its route with the full marathon (**Figure 2**). Although both events follow major urban roads during the first half of the race, the half-marathon remains within the city street network during the latter half and does not include the Keelung River waterfront sections traversed by full-marathon runners. Along the course, water or sponge stations are placed at approximately 5-km intervals. For the full marathon, there are 8 water/drink stations (at around 5, 10, 15, 20, 25, 30, 35, and 40 km) and 7 sponge stations located between them. For the half-marathon, there are 4 water/drink stations (at approximately 5, 10, 15, and 20 km) and 3 sponge stations positioned between the water stations. The full marathon commences at 6:30 a.m. (7:00 a.m. in 2013 and 2014), with a cut-off time of 5 h 30 min. The half-marathon starts at 7:00 a.m. (6:30 a.m. in 2015), with a cut-off time of 3h (3 h 30 min in 2013 and 2014).

**Figure 1:**
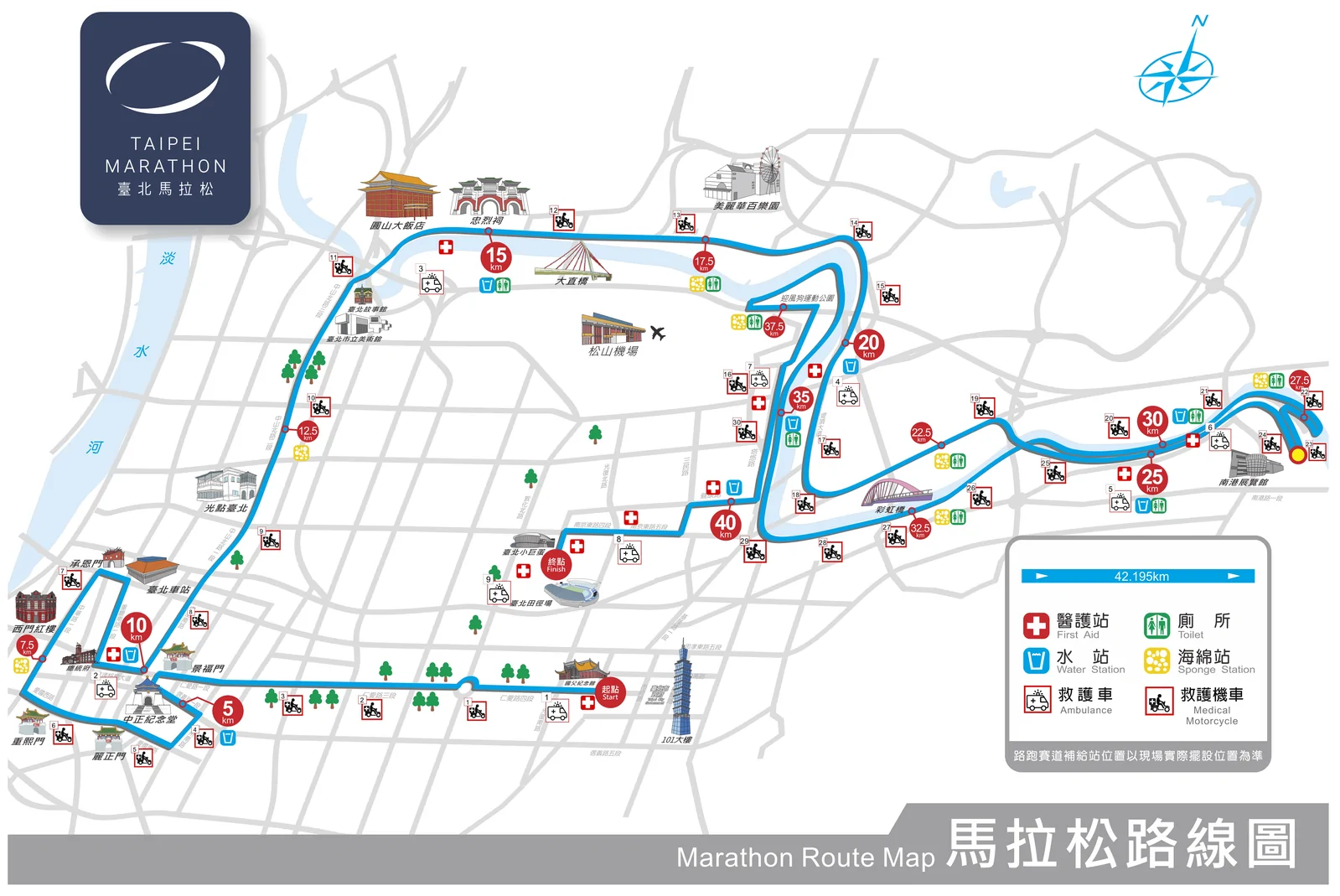
Full marathon course route and medical service locations for the Taipei Marathon.

**Figure 2:**
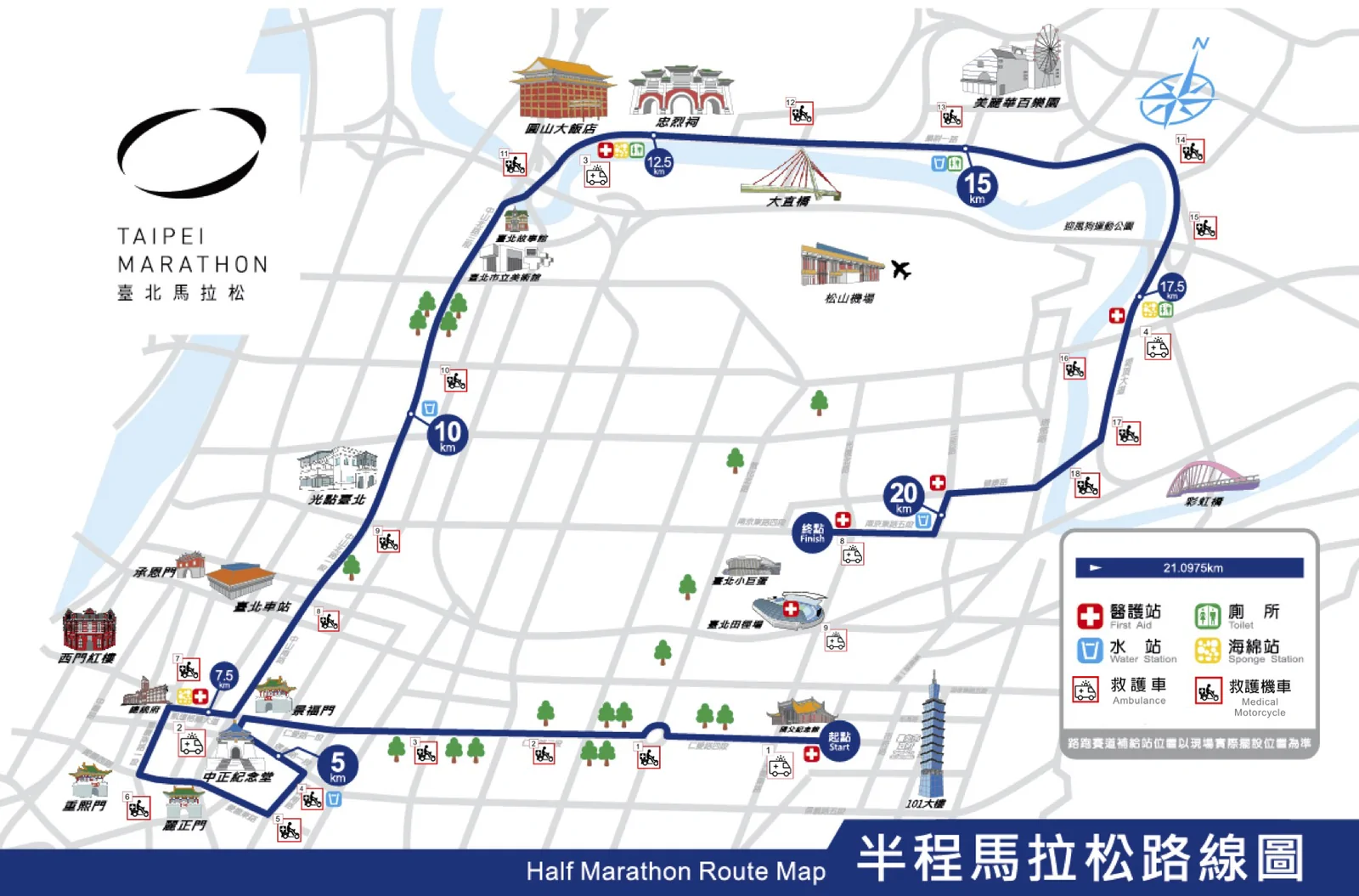
Half-marathon course route and medical service locations for the Taipei Marathon.

A retrospective analysis was conducted using a database provided by the Association of Emergency Medical Services at Mass Gatherings (AEMSMG), Taiwan, R.O.C., which has been responsible for planning and providing onsite emergency medical care for this event since 2001. Ethical approval was obtained from the MacKay Memorial Hospital Institutional Review Board (22MMHIS027e).

### 2.2 Meteorological data

Weather data for the period from 7 days before each marathon event through the race day (2013–2024) were obtained from the Central Weather Bureau of Taiwan (**Figure 3**), with measurements matched to the event locations in Taipei City and race hours (06:00–12:00).

**Figure 3:**
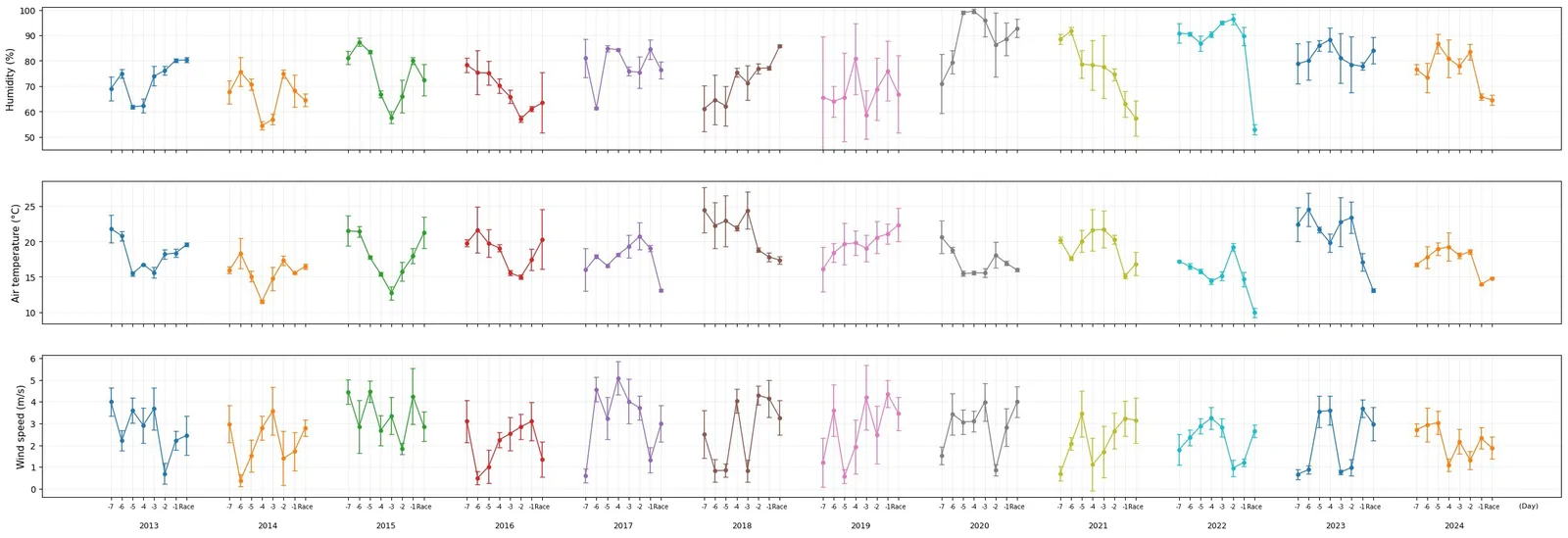
Meteorological data, including air temperature, relative humidity, and wind speed, were obtained from the Central Weather Bureau of Taiwan for the period spanning 7 days before each marathon event through race day between 2013 and 2024.

AT, an index integrating the effects of air temperature, humidity, and wind speed on perceived thermal stress, was calculated according to Steadman’s formula (Steadman, 1984):


AT=Ta+0.33×e−0.70×ws−4.00

where:

Ta = Dry bulb temperature (°C).
e = Water vapor pressure (hPa) [humidity].
ws = Wind speed (m/s) at an elevation of 10 m.

Water vapor pressure was calculated from the temperature and relative humidity using the following equation:


e=rh/100×6.105×exp(17.27×Ta/(237.7+Ta))

where:

rh = Relative Humidity [%].

To better characterize the association between pre-race AT variability and the incidence rate of SCA on race days, we calculated the SD of AT over multiple pre-race time windows, ranging from Lag 0–1 to Lag 0–7. Specifically, the SD of AT for Lag 0–1 was calculated using AT values on race days (lag 0) and 1 day before the race (lag 1). The SD of AT for Lag 0–2 was calculated using AT values from race days and the preceding 2 days, and this approach was extended sequentially through Lag 0–7.

### 2.3 EMS at the marathon

The AEMSMG, Taiwan, R.O.C., organizes and implements EMS in a consistent manner each year. Ten medical tents, one first-aid station, and nine event ambulances are distributed along the course of the event, and one medical tent is placed near the baggage storage area. The medical director is responsible for medical supervision and remotely overseeing the overall functioning of the medical units in a medical command center that is set up 100 m from the finish line. In Taipei Marathon events, most of the half-marathon course overlaps with that of the full marathon. Each medical tent comprised one doctor, one to three nurses, and one to three paramedics. Besides fixed medical tents, the in-event EMS also comprised a mobile first-aid team. Thirty paramedics rode motorcycles and carried automated external defibrillators (AEDs) and emergency medical kits throughout the racecourse. Paramedics who provided medical services on the first one-third of the racecourse moved to the latter part of the course as the contestants moved ahead.

### 2.4 Statistical analysis

Descriptive results are reported as median (interquartile range [IQR]). Poisson regression models with the logarithm of the number of participants included as an offset were used to assess the association between the SD of AT across different cumulative lag windows and the incidence rate of SCA. Poisson regression models were fitted with the number of SCA events as the outcome and the logarithm of the number of race participants included as an offset, thereby modeling the incidence rate of SCA. The models were adjusted for participants’ mean age, race-day air temperature, relative humidity, and wind speed. Incidence rate ratios (IRRs) and their 95% profile likelihood confidence intervals (CIs) were estimated. Statistical significance was assessed using likelihood ratio tests (LRTs), with a two-sided p value < 0.05 considered statistically significant. Model fit was evaluated using the Akaike information criterion (AIC), and the Pearson dispersion ratio was examined to assess overdispersion. Statistical analyses were performed using R version 4.6.1 (R Foundation for Statistical Computing, Vienna, Austria).

## 3 Results

The total number of participants in the Taipei Marathon events between 2013 and 2024 was 316,446, comprising 95,866 marathoners and 220,580 half-marathoners. The total number of male runners was 232,020, while the number of female runners was 84,426. The average number of participants was 26,371 per year (approximately 7989 marathoners and 18,382 half-marathoners).

### 3.1 Demographic analysis

Thirteen Taiwanese runners experienced SCA, including 12 men and 1 woman. The incidence was higher in men (5.17 per 100,000; 95% confidence interval [CI] 2.60–9.03) than in women (1.18 per 100,000; 95% CI 0.03–6.60). The median age of the participants with SCAs was 42 years (range, 24–60 years), whereas the median annual estimated mean age of all participants without SCA was 39.6 years. Among the 13 cases, 3 occurred during full marathons and 10 during half-marathons. The incidence of SCA was higher during half-marathons (4.53 per 100,000; 95% CI 2.17–8.34) compared with full marathons (3.13 per 100,000; 95% CI 0.65–9.15). The annual incidence ranged from 0–1.93 per 10,000 participants, with rates of 0–2.15 and 0–2.76 per 10,000 participants in full- and half-marathons, respectively.

### 3.2 Weather effects

The associations between the SD of AT calculated over different cumulative lag windows and the incidence rate of SCA are shown in **Figure 4**. Statistically significant positive associations were observed for Lag 0–2 through Lag 0–7, whereas no significant association was observed for Lag 0–1. For the Lag 0–6 window, each 1 °C increase in the SD of AT was associated with an SCA incidence rate that was 6.63 times as high on race day (IRR = 6.63, 95% profile likelihood CI 1.85–52.11; LRT p < 0.001).

**Figure 4:**
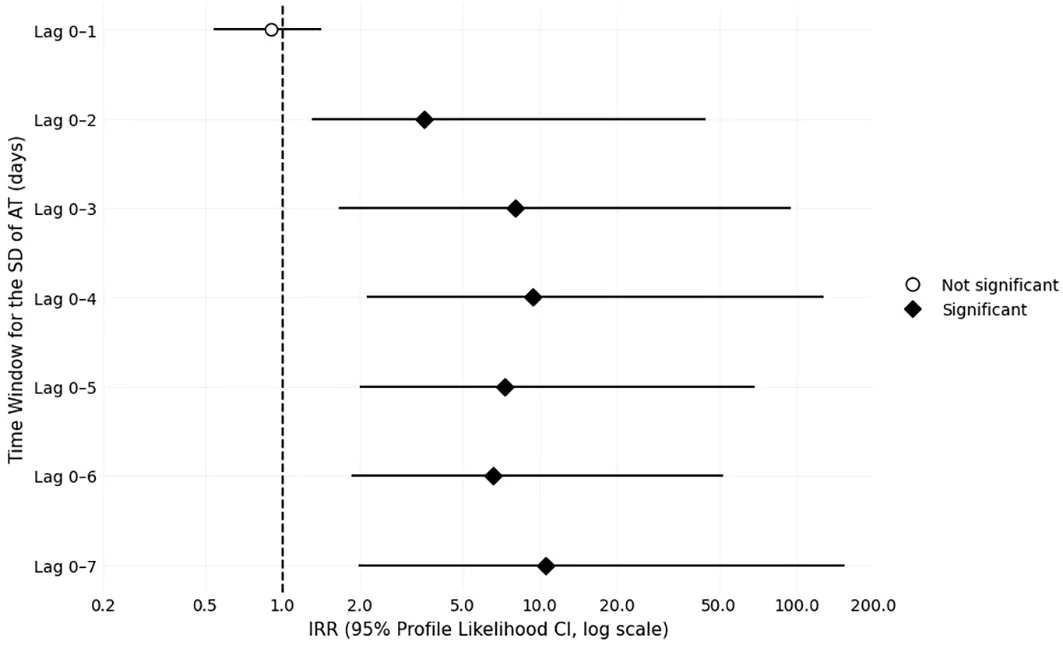
Association between apparent temperature (AT) variability and the incidence rate of sudden cardiac arrest during Taipei Marathon events. Incidence rate ratios (IRRs) and 95% profile likelihood confidence intervals (CIs) are shown for the standard deviation (SD) of AT calculated over different time windows (Lag 0–1 to Lag 0–7). Models were adjusted for participants’ mean age, race-day air temperature, relative humidity, and wind speed. IRRs are presented on a logarithmic scale. The dashed vertical line represents an IRR of 1; open circles indicate non-significant associations and filled diamonds indicate statistically significant associations based on likelihood ratio tests.

### 3.3 EMS response

Regarding the location of SCA cases, 8 (61.5%) occurred in the final quarter of the race (Q4), 3 (23.1%) in the third quarter (Q3), and 1 (7.7%) each in the second (Q2) and first (Q1) quarters. Details about the EMS response are shown in **Table 1**. All SCA events (100%) were witnessed, and bystander-administered cardiopulmonary resuscitation (CPR) was provided in every case. The median duration of the low-flow or no-flow state was 1 min (range, 0–3 min). AED shocks were delivered to 11 of the 13 runners (84.6%) at a median 3 min after collapse (range, 1–7 min), including 9 runners with ventricular fibrillation and 2 with ventricular tachycardia. The remaining two runners (Cases 1 and 8) experienced cardiac arrest, as evidenced by the absence of a palpable carotid pulse. Both runners achieved a return of spontaneous circulation (ROSC) following CPR alone before an AED became available. Therefore, the initial cardiac rhythm could not be determined. All 13 runners with SCA (100%) achieved ROSC in the field. Of these, 12 (92.3%) remained alive at 24 h and survived to hospital discharge or 30-day follow-up.

**Table 1:** Demographic characteristics and emergency medical services among runners with cardiac arrests (N = 13).

Case	Event	Age (year)	Gender	Time from race start to SCA (min)	Location	Bystander-administered CPR	Time from collapse to CPR initiation (min)	Time from collapse to AED shock delivery (min)	AED report	ROSC in the field	24-hour survival	Survival at discharge	30-day survival
1	2013Half-marathon	60	M	200	Q4	Yes	0.5	–	–	Yes	No	No	No
2	2014 Half-marathon	50	M	158	Q4	Yes	0.5	2	Vf	Yes	Yes	Yes	Yes
3	2015 Marathon	50	M	200	Q3	Yes	3	4	Vf	Yes	Yes	Yes	Yes
4	2017 Half-marathon	27	M	170	Q4	Yes	1	2	Vf	Yes	Yes	Yes	Yes
5	2021 Half-marathon	26	M	70	Q2	Yes	1	7	Vf	Yes	Yes	Yes	Yes
6	2021 Half-marathon	33	M	132	Q4	Yes	1	6	VT	Yes	Yes	Yes	Yes
7	2021 Half-marathon	54	M	88	Q3	Yes	0	2	Vf	Yes	Yes	Yes	Yes
8	2021 Half-marathon	24	F	176	Q4	Yes	1	–	–	Yes	Yes	Yes	Yes
9	2021 Half-marathon	27	M	166	Q4	Yes	0	1	Vf	Yes	Yes	Yes	Yes
10	2022 Half-marathon	42	M	126	Q4	Yes	2	3	VT	Yes	Yes	Yes	Yes
11	2023 Half-marathon	26	M	108	Q4	Yes	0	1	Vf	Yes	Yes	Yes	Yes
12	2023 Marathon	50	M	166	Q3	Yes	1	6	Vf	Yes	Yes	Yes	Yes
13	2023 Marathon	46	M	45	Q1	Yes	2	4	Vf	Yes	Yes	Yes	Yes

AED, automated external defibrillator; CPR, cardiopulmonary resuscitation; ROSC, return of spontaneous circulation; SCA, sudden cardiac arrest. Vf, ventricular fibrillation; VT, ventricular tachycardia. Q1 denotes 0 to 5.28 km, Q2 5.28 to 10.55 km, Q3 10.55 to 16 km, and Q4–16 km to finish in half-marathon.

Q1 denotes 0 to 10.55 km, Q2 10.55 to 21 km, Q3–21 to 32 km, and Q4–32 km to finish in marathon.

## 4 Discussion

Direct evidence linking cold weather to marathon-related SCA remains limited; however, our findings raise the possibility that an elevated incidence of SCA may be associated with substantial temperature fluctuations during winter. Interestingly, while non-Taiwanese runners accounted for approximately 4–16% of participants in the Taipei Marathon across most study years, all recorded cases of cardiac arrest occurred exclusively among Taiwanese runners. International participation was negligible in 2020 and 2021, primarily attributable to coronavirus disease (COVID-19) pandemic restrictions and Taiwan’s border control measures. This observation should be interpreted with caution given the small number of events. Nevertheless, our study provides valuable insights for marathon participants, coaches, and medical providers regarding the potential impact of temperature variability on the occurrence of SCA during city marathons, as well as the importance of prompt CPR and rapid defibrillation.

Participation in winter sports may pose unique cardiovascular challenges due to the combined effects of environmental and exercise-related stressors. Kato et al. reported a nationwide Japanese registry study showing a non-linear relationship between air temperature and exercise-related SCA in school-aged children and adolescents, with the risk increasing in colder environmental conditions (Kato et al., 2025). Some postulated mechanisms could explain the risk of cardiovascular disease mortality associated with lower temperatures. Cold exposure leads to increased sympathetic activity, which vasoconstricts the skin and increases skeletal muscle tone to ensure core body thermal homeostasis. This leads to a catecholamine-driven rise in blood pressure and, hence, increased cardiac oxygen demand to overcome this increase in afterload. Cold also leads to increased cholesterol crystal deposition in atherosclerotic plaques, rendering individuals susceptible to plaque rupture and myocardial infarction. Hypothermia is known to induce hypercoagulability due to increased viscosity and hemoconcentration from fluid shifting into the extravascular space and clotting factor abnormalities (Stewart et al., 2017; Alahmad et al., 2023). Marathon running increases heart rate and myocardial oxygen demand, whereas exposure to cold weather elevates systemic vascular resistance and blood pressure. The combination of strenuous exercise and cold exposure may therefore act synergistically to increase cardiovascular stress and potentially raise the risk of SCA in susceptible individuals.

Importantly, SCA during marathon running is rare, and most cases are attributable to underlying structural or electrical cardiac disease rather than thermoregulatory failure alone (Kim et al., 2012, Kim et al., 2025). In a cohort study from the Race Associated Cardiac Event Registry (RACER), the incidence of SCA during US marathons and half-marathons was 0.54 per 100,000 participants in the 2000–2009 analyses and 0.60 per 100,000 participants in the 2010–2023 analyses (Kim et al., 2012, Kim et al., 2025). The Registre des Accidents Cardiaques lors des courses d’Endurance à Paris (RACE PARIS registry) recorded the incidence of SCA during marathons, half-marathons, and other long-distance races in the Paris area between 2006 and 2016 is 1.67 per 100,000 participants (Gerardin et al., 2016, Gerardin et al., 2021). During intense endurance exercise, trained athletes generate substantial metabolic heat, and core body temperature can rise to ~38.5–40 °C or higher in prolonged events, particularly in hot or humid environments (Singh et al., 2023). Even in well-trained individuals with efficient thermoregulation, dehydration, reduced skin blood flow, and environmental heat load can impair heat dissipation. This leads to increased sympathetic activation, tachycardia, reduced plasma volume, and heightened cardiovascular strain (Périard et al., 2021). As the Taipei Marathon is held annually during winter under cool and high-humidity conditions, with an air temperature range of 9.1–26 °C and relative humidity of 45–96% (AT range: 5.2–25.7 °C) during the study period, heat-related disorders may still occur and may potentially contribute to cardiovascular collapse. Beyond the effects of absolute temperature, accumulating epidemiological evidence suggests that short-term temperature variability over several days may independently contribute to cardiovascular stress and influence mortality risk (Keatinge et al., 1984; Hanneman et al., 2026). In line with documented lagged associations between temperature exposure and cardiovascular outcomes, we observed positive associations between AT variability and SCA incidence across longer exposure windows (lag 0–2 through lag 0–7); however, no significant association emerged for lag 0–1. These findings suggest that cumulative short-term AT variability over several days preceding and including race day may influence SCA susceptibility during marathons. The consistent associations observed across longer exposure windows, including the lag 0–6, may indicate that approximately 1 week timeframe represents a critically relevant exposure window for AT variability. However, given the limited number of SCA events and correspondingly wide confidence intervals, these findings must be interpreted cautiously and require confirmation using larger datasets.

Men, especially those over 40 years of age, are at a higher risk of cardiac events during marathons than women. Several mechanisms may contribute to this sex-specific divergence, including the higher prevalence of occult coronary artery disease in middle-aged men, sex-related variations in ventricular arrhythmia susceptibility, and the cardioprotective effects of estrogen in women (Kim et al., 2012; Roberts et al., 2013; Li et al., 2025). Findings from RACER showed that the proportion of cardiac arrests among male full-and half-marathon runners was 1.12 per 100,000, compared with 0.19 per 100,000 among women (Kim et al., 2025). Our results were similar to those described above. The incidence of SCA was markedly lower in female runners than in male runners. In both the RACER dataset and the RACE Paris registry, SCAs were more likely to occur in the last quartile of races, when runners often increased their intensity as they approached the finish line (Gerardin et al., 2016, Gerardin et al., 2021; Kim et al., 2025). In our study, SCAs occurred over the entire race length. Most SCAs occurred in the last quarter of the race (Q4), followed by the third quarter (Q3), which is consistent with previous literature. The RACER database of marathon and half-marathon races in the United States held in 2000–2010 concluded that the incidence of SCA was significantly higher during marathons (1.01 per 100,000) than during half-marathons (0.27 per 100,000) (Kim et al., 2012). For the follow-up study period (2010–2023), cardiac arrest risk at the marathon distance remains higher during marathons (1.04 per 100,000) compared with half-marathons (0.47 per 100,000) (Kim et al., 2025). However, the RACE PARIS registry that enrolled Paris races (Paris marathon, Paris and Boulogne half-marathon, Paris 20-km race, and the Paris-Versailles 16.2-km race) held in 2006–2016 reported a higher incidence of SCA in half-marathon or equivalent runners (1.9 per 100,000) than marathoners (1.1 per 100,000) (Gerardin et al., 2016, Gerardin et al., 2021). The incidence of SCA in the present study is consistent with Gerardin’s findings, showing a higher rate in half-marathoners (4.53 per 100,000) than in marathoners (3.13 per 100,000). One plausible explanation is that half-marathons attract a larger proportion of recreational or less-experienced runners, who may have exhibit suboptimal levels of endurance training than experienced full-marathon runners.

Using RACER data, Kim et al. reported a slight increase in the incidence proportion of cardiac arrests in 2020–2023 (Kim et al., 2025). Consistent with the RACER data, Taipei Marathons recorded a significantly higher incidence of SCAs between 2020 and 2023 (8.14 per 100,000 participants) than between 2013 and 2019 (2.25 per 100,000 participants). The reasons for this slight increase in the incidence of cardiac arrests after 2020 remain unknown. However, the COVID-19 pandemic and the associated public health measures, such as social distancing and movement restrictions, likely had a substantial impact on mass-gathering sporting events. Several studies have revealed that the pandemic has changed the physical activity of many individuals and the training habits of athletes (Castañeda-Babarro et al., 2020; Di Corrado et al., 2020). DeJong showed a tendency of decreasing running intensity in runners during the COVID-19 pandemic, and Matsumura et al. also reported that the COVID-19 pandemic had heterogeneous effects on the fitness of high-level non-elite runners (DeJong et al., 2021; Matsumura et al., 2021). In Taiwan, the government announced COVID-19-related restrictions from May 15 to July 26, 2021. These prohibited the opening of sports centers and outdoor gatherings, to comply with the need for social distancing as previously mentioned. The 2021 Taipei Marathon event was the first mass-gathering sporting event that took place after the COVID-19 high-alert status and its related restrictions were lifted. During the early stages of the COVID-19 pandemic, Taiwan experienced a relatively low number of severe acute respiratory syndrome coronavirus-2 (SARS-CoV-2) infections (2020: cumulative incidence, 3.49 per 100,000 population; 2021: cumulative incidence, 69.75 per 100,000 population) (Lai et al., 2023; Chen and Huang, 2024). In 2022, Taiwan began reporting imported cases of the COVID-19 Omicron variant. These cases led to multiple community-cluster outbreaks, eventually resulting in a large-scale COVID-19 epidemic (Su, 2023). Although concerns have been raised regarding potential cardiovascular complications following SARS-CoV-2 infection, myocarditis in competitive athletes after infection appears to be uncommon (Kim et al., 2021; Moulson et al., 2021; Kim et al., 2025). COVID-related factors may therefore have contributed to the higher SCA incidence observed after 2020; however, our study was not designed to disentangle the effects of the pandemic from those of temperature variability. Therefore, we cannot determine the extent to which the higher SCA incidence observed in 2021 and 2023 was attributable to the COVID-19 pandemic versus AT variability.

The provision of in-event emergency care is subject to variations in organization among EMS providers, with domestic and regional as well as international differences. A retrospective study by Robers on American marathons recommends setting up fixed medical stations at 2- to 3-mile intervals, and mobile AED-equipped first-aid teams throughout the racecourse (Robers and Maron, 2005). A review by Day suggests that trained AED-equipped personnel every 200–300 m or a large number of mobile rapid-response patrols are required to achieve the goal of defibrillation within 1–2 min from cardiac arrest onset (Day and Thompson, 2010). Kinoshi proposed a rapid mobile AED system for SCA runners during road races. Their system consists of mobile teams on bicycles with AEDs and emergency medical kits, and on-foot teams who carry AEDs providing basic life support (Kinoshi et al., 2018). The EMS resources required for marathons are likely to vary according to race course, such as route configuration (e.g., an out-and-back course), the elevation profile, and other course-specific features. Based on the observed pattern of EMS utilization, we deployed additional medical tents and event ambulances on the latter race course (Chan et al., 2019; Parker et al., 2021). In our system, there was one mobile AED-equipped paramedic at 1.2–1.4-km intervals within the first 20 km of the full-marathon course and the first 17.5 km of the half-marathon course. As mentioned earlier, 12 mobile paramedics riding motorcycles who initially serviced the first one-third of the marathon course successively shifted to the latter one-third as the participants moved ahead. As such, there was an average of one mobile paramedic per 0.88 km between the 20-km mark and the finishing point for the full marathon. For the half-marathon, there was an average of one mobile paramedic per 0.7 km in the last 3.5 km of the course. Therefore, such EMS intensity could provide SCA runners with effective and timely resuscitation. In addition, fellow runners are often the first responders to assist a collapsed runner during a race. Rapid initiation of bystander-initiated CPR and defibrillation are essential factors for survival (Ewert, 2007; Johnston et al., 2019). The use of digital and personalized systems could increase the survival of out-of-hospital cardiac arrest by accelerating the first responder dispatch and realizing the public health potential of bystander-initiated CPR (Derkenne et al., 2020). From our data, all runners with SCA (100%) achieved ROSC at the scene. There was only one death within 24 h. The low case fatality rate (7.7%) is consistent with a previous report of the RACE PARIS registry (case fatality rate, 11.1%) (Gerardin et al., 2021). This implies that bystanders can play a critical role in improving survival rates when they are trained to start CPR and call for help.

Our study is among the few to examine relationship between temperature fluctuations and SCA risk in marathoners. The Taipei Marathon events feature minimal course variation and are held in a consistent season (December) to ensure stable environmental and course conditions that facilitate year-to-year comparisons. We used integrated medical records provided by the AEMSMG, the organization responsible for planning medical stations and delivering on-site EMS during the study period. The team employed standardized outcome measures to minimize the potential for misclassification. The present study has some limitations. First, our analysis was constrained by the relatively short study period and the limited number of events, which may have compromised statistical power and estimate precision, precluding definitive conclusions. Although weather data from national monitoring stations were used, measurement errors may have occurred. Importantly, weather data for the period from 7 days before each marathon event through race day were obtained with measurements matched to the event locations in Taipei City and race hours. Therefore, the weather fluctuations analyzed in this study reflect conditions at the marathon venues during the periods when the events were conducted. However, these ecological data were not available at the individual level for runners who experienced SCA during the events and may not accurately represent their actual environmental exposure. Furthermore, we did not account for individual differences in temperature exposure arising from variations in race duration or exercise intensity.

## 5 Conclusion

Greater short-term variability in AT during the 06:00–12:00 period was associated with an increased incidence of reported SCA during the Taipei Marathon events across exposure windows ranging from the preceding 2 to 7 days. These findings suggest that short-term AT variability over several days preceding and including race day may be relevant to the occurrence of SCA during winter marathons. Runners should implement timely prevention strategies during periods of low temperature. Measures to reduce the risk of both cold- and heat-related conditions that may contribute to cardiovascular arrest should include adequate acclimatization, pre-participation medical screening, and the use of appropriate race-day clothing. The on-site presence of trained first responders to initiate CPR is mandatory at these events. Medical providers must be more precise and anticipate the risk at the last stage of the race where the majority of SCAs occur. Since these SCAs occurred during the city marathon, the combination of in-event and city EMS allowed the chain of survival to work smoothly.

## Data Availability

The original contributions presented in the study are included in the article/supplementary material. Further inquiries can be directed to the corresponding authors.
